# Supplementary material for: Unveiling the cut-and-repair cycle of designer nucleases in human stem and T cells via CLEAR-time dPCR
Source: Nat Commun. 2025 Nov 3;16:9571. doi: 10.1038/s41467-025-65182-4 (PMC12583642; doi:10.1038/s41467-025-65182-4)
Supplement: Supplementary file 1 — Supplementary Information [file 41467_2025_65182_MOESM1_ESM.pdf]

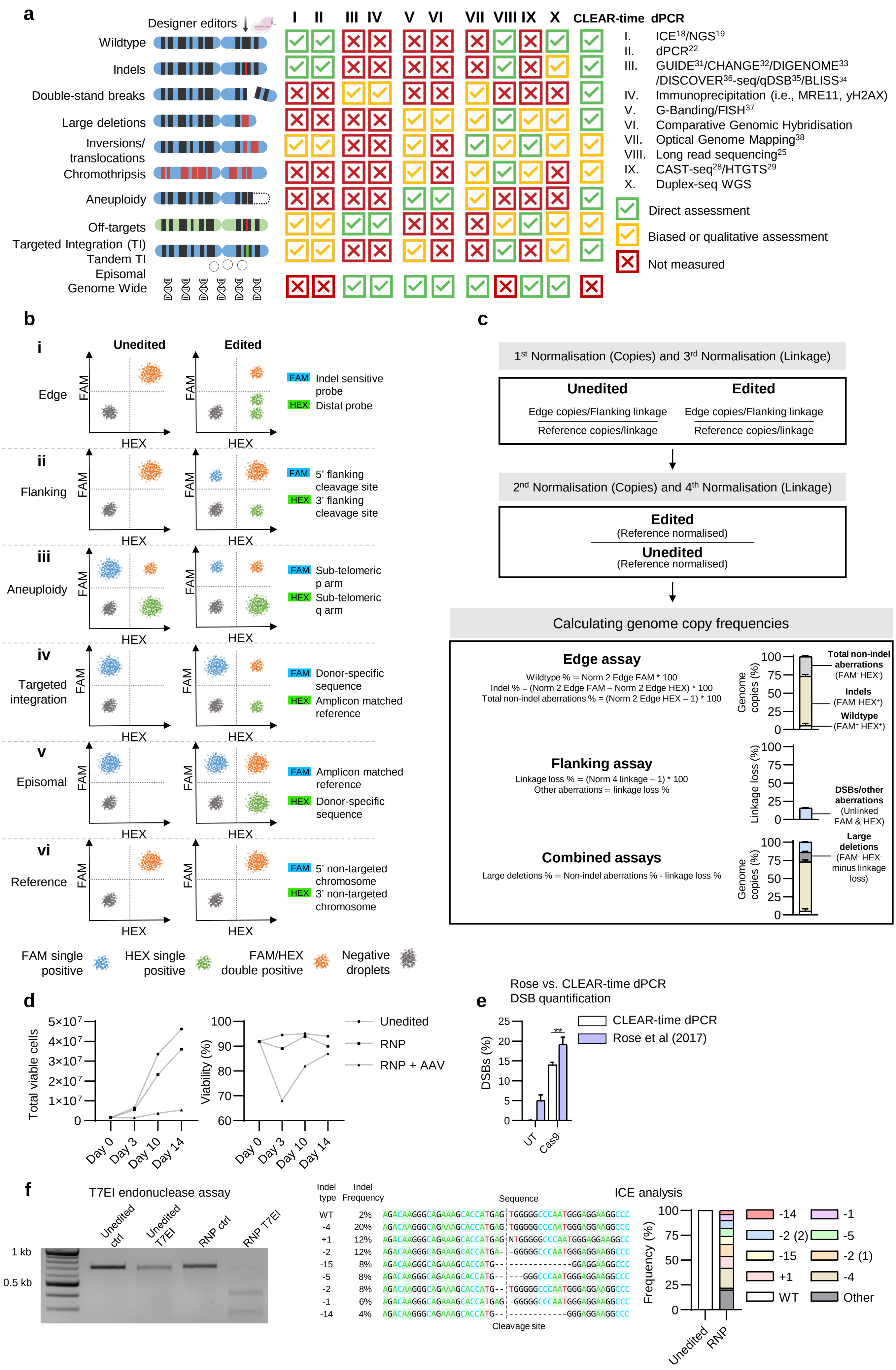
a
Designer editors
I II III IV V VI VII VIII IX X CLEAR-time dPCR
Wildtype
Indels
Double-stand breaks
Large deletions
Inversions/ translocations
Chromothripsis
Aneuploidy
Off-targets
Targeted Integration (TI)
Tandem TI
Episomal
Genome Wide
I. ICE18/NGS19
II. dPCR22
III. GUIDE31/CHANGE32/DIGENOME33/DISCOVER36-seq/qDSB35/BLISS34
IV. Immunoprecipitation (i.e., MRE11, yH2AX)
V. G-Banding/FISH37
VI. Comparative Genomic Hybridisation
VII. Optical Genome Mapping38
VIII. Long read sequencing25
IX. CAST-seq28/HTGTS29
X. Duplex-seq WGS
Direct assessment
Biased or qualitative assessment
Not measured
b
Unedited
Edited
i Edge
FAM Indel sensitive probe
HEX Distal probe
ii Flanking
FAM 5' flanking cleavage site
HEX 3' flanking cleavage site
iii Aneuploidy
FAM Sub-telomeric p arm
HEX Sub-telomeric q arm
iv Targeted integration
FAM Donor-specific sequence
HEX Amplicon matched reference
v Episomal
FAM Amplicon matched reference
HEX Donor-specific sequence
vi Reference
FAM 5' non-targeted chromosome
HEX 3' non-targeted chromosome
FAM HEX
FAM single positive
HEX single positive
FAM/HEX double positive
Negative droplets
c
1st Normalisation (Copies) and 3rd Normalisation (Linkage)
Unedited
Edited
Edge copies/Flanking linkage
Reference copies/linkage
2nd Normalisation (Copies) and 4th Normalisation (Linkage)
Edited (Reference normalised)
Unedited (Reference normalised)
Calculating genome copy frequencies
Edge assay
Wildtype % = Norm 2 Edge FAM * 100
Indel % = (Norm 2 Edge FAM – Norm 2 Edge HEX) * 100
Total non-indel aberrations % = (Norm 2 Edge HEX – 1) * 100
Flanking assay
Linkage loss % = (Norm 4 linkage – 1) * 100
Other aberrations = linkage loss %
Combined assays
Large deletions % = Non-indel aberrations % - linkage loss %
Genome copies (%)
Linkage loss (%)
Total non-indel aberrations (FAM- HEX-)
Indels (FAM- HEX+)
Wildtype (FAM+ HEX+)
DSBs/other aberrations (Unlinked FAM & HEX)
Large deletions (FAM- HEX- minus linkage loss)
d
Total viable cells
Viability (%)
Day 0 Day 3 Day 10 Day 14
Unedited
RNP
RNP + AAV
e
Rose vs. CLEAR-time dPCR DSB quantification
DSBs (%)
CLEAR-time dPCR
Rose et al (2017)
UT Cas9
**
f
T7EI endonuclease assay
Unedited ctrl
Unedited T7EI
RNP ctrl
RNP T7EI
1 kb
0.5 kb
Indel type
Indel Frequency
Sequence
WT 2%
-4 20%
+1 12%
-2 12%
-15 8%
-5 8%
-2 8%
-1 6%
-14 4%
Cleavage site
ICE analysis
Frequency (%)
Unedited RNP
-14 -1 -2 (2) -5 -15 -2 (1) +1 -4 WT Other

Supplementary Figure 1 | CLEAR-time dPCR assays and indel validation

(**a**) Table schematic of the capabilities of various technologies used to identify and quantify designer nuclease induced aberrations. (**b**) Schematic of dPCR expected dot plots and thresholding logic of unedited and edited cells for each mutation (i-v) and refence (vi) CLEAR-time dPCR assay. (**c**) Double normalisation and genome copy frequency calculation workflow. (**d**) Total viable CD34+ HSPC counts and viability percentage of unedited, RNP, and RNP + AAV HSPCs on the day of editing, 3-, 10-, and 14-days post-editing. n=2 technical replicates. Related to Fig. 1c. (**e**) Comparison of CLEAR-time dPCR DSB quantification with previously published dPCR method. (**f**) Indel frequency assessment of RNP only edited cells using T7EI endonuclease assay and relative quantification of indel populations with ICE analysis 3 days post-editing. Indels from T7EI assay were calculated as follows: $\text{Indel}\ \% = \frac{\text{Digested products}}{(\text{Digested products} + \text{Undigested band})}$. Related to Fig.1g.

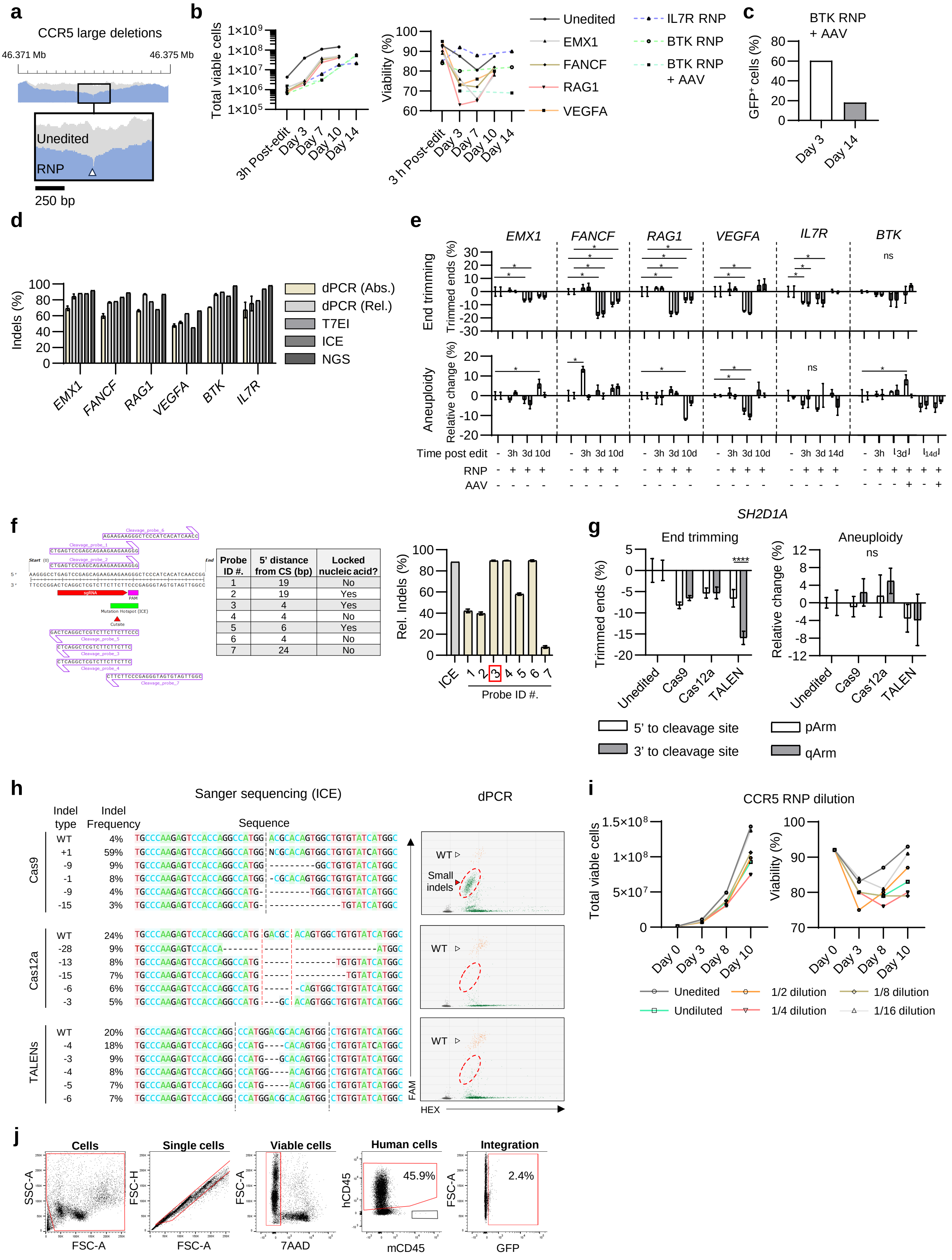

Probe ID #.	5' distance from CS (bp)	Locked nucleic acid?
1	19	No
2	19	Yes
3	4	Yes
4	4	No
5	6	Yes
6	4	No
7	24	No

Supplementary Figure 2 | CLEAR-time dPCR applications

(**a**) NGS targeted sequencing spanning ~2500 bp of the cleavage site targeting *CCR5* (white arrowhead) indicating small and large deletions (> 250 bp). X-axis indicates nucleotide position; Y-axis indicates number of mapped reads. Scale bar indicates 250 bp. (**b**) Comparison of unedited, RNP, and RNP + AAV total viable cell counts and viability frequency for HSPCs edited at *EMX1*, *FANCF*, *RAG1*, *VEGFA*, *IL7R*, and *BTK* loci 3-hours, 3-, 7-, 10-, and 14-days post-editing. (n=2 technical replicates; related to Fig. 1c). (**c**) Flow cytometry quantification of GFP+ AAV-transduced cells at *BTK* locus 3- and 14-days post-editing; related to Fig. 2c. (**d**) Validation of indel frequency at various loci by comparing the relative indel frequency calculated by dPCR, T7EI assay, ICE, and NGS analysis measured in HSPCs 3-days post-editing. Related to Fig. 2c. (**e**) End trimming and aneuploidy dPCR summaries of Cas9 edited HSPCs targeting various genes at 3 hours, 3- and 10-days post-editing. Data are shown as mean ± s.d. (n=3 technical replicates; related to Fig. 2c). Two-way ANOVA with Tukey multiple comparisons test. *p<0.05. (**f**) Optimisation of Edge cleavage probe placement around the cleavage site (left) and comparison of relative indel quantification to sequencing (right). Red box indicates EMX1 cleavage probe used in subsequent experiments. (**g**) Comparison of Cas9, Cas12a, and TALEN nuclease generated end trimming and aneuploidy in T cells at the *SH2D1A* loci 3-days post-editing. Data are shown as mean ± s.d. Two-way ANOVA with Sidak's multiple comparison test. ****p<0.0001. (n=3 technical replicates; related to Fig. 2d). (**h**) Comparison of relative quantification of indel generated by Cas9, Cas12a, or TALENs nuclease using ICE analysis at the *SH2D1A* loci 3-days post-editing. Representative dPCR dot-plots of edge assay illustrating intermediate likely small indel population of double-positive droplets (red arrowhead) below the wildtype population (white arrowhead) present only in Cas9 edited T cells. Single black dotted, double red dotted, and double black dotted lines represent Cas9 cleavage site, staggard Cas12a cleavage sites, and TALENs cleavage window, respectively. Related to Fig. 2d. (**i**) Comparison of viable cell counts and viability frequency between unedited, and diluted RNP edited HSPCs at the *CCR5* loci 3-days post-editing. (n=2 technical replicates). Related to Fig. 2e, f. (**j**) Representative flow cytometry gating strategy used for quantifying GFP+ in hCD45 enriched cells from murine bone marrow. Red boxes indicate gating position. Related to Fig. 2g.

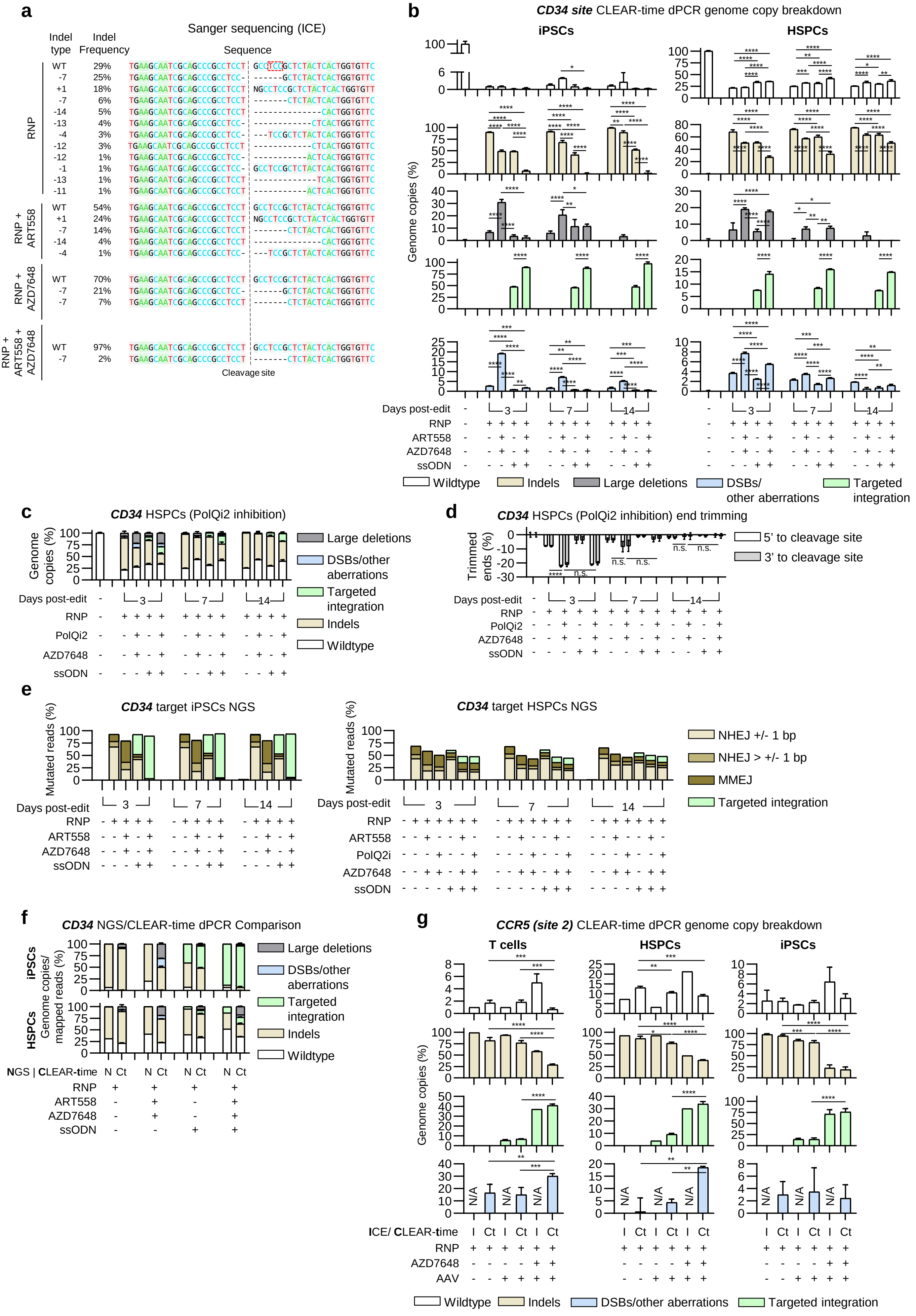
a
Sanger sequencing (ICE)
Indel type
Indel Frequency
Sequence
RNP
RNP + ART558
RNP + AZD7648
RNP + ART558 + AZD7648
Cleavage site
b
CD34 site CLEAR-time dPCR genome copy breakdown
iPSCs
HSPCs
Genome copies (%)
Days post-edit
RNP
ART558
AZD7648
ssODN
Wildtype
Indels
Large deletions
DSBs/ other aberrations
Targeted integration
c
CD34 HSPCs (PolQi2 inhibition)
Genome copies (%)
Days post-edit
RNP
PolQi2
AZD7648
ssODN
Large deletions
DSBs/other aberrations
Targeted integration
Indels
Wildtype
d
CD34 HSPCs (PolQi2 inhibition) end trimming
Trimmed ends (%)
5' to cleavage site
3' to cleavage site
Days post-edit
RNP
PolQi2
AZD7648
ssODN
e
CD34 target iPSCs NGS
CD34 target HSPCs NGS
Mutated reads (%)
Days post-edit
RNP
ART558
PolQ2i
AZD7648
ssODN
NHEJ +/- 1 bp
NHEJ > +/- 1 bp
MMEJ
Targeted integration
f
CD34 NGS/CLEAR-time dPCR Comparison
iPSCs
HSPCs
Genome copies/ mapped reads (%)
Large deletions
DSBs/other aberrations
Targeted integration
Indels
Wildtype
NGS | CLEAR-time
RNP
ART558
AZD7648
ssODN
g
CCR5 (site 2) CLEAR-time dPCR genome copy breakdown
T cells
HSPCs
iPSCs
Genome copies (%)
ICE/ CLEAR-time
RNP
AZD7648
AAV
Wildtype
Indels
DSBs/other aberrations
Targeted integration

Supplementary Figure 3 | Targeted integration enhancers

(**a**) Comparison of relative quantification of indels generated by RNP, RNP + ART558, RNP + AZD7648, and RNP + ART558 + AZD7648 through ICE analysis at the *CCR5* locus 1-day post-editing. Red-dotted box indicates microhomology region. Related to Fig. 3a.

(**b**) CLEAR-time dPCR genome copy summary breakdown of aberrations induced by repair inhibitors in Cas9 edited iPSCs (left) and HSPCs (right) targeting the *CD34* locus with and without an ssODN 3, 7, and 14-days post-editing. Related to Fig. 3c. (**c**) CLEAR-time dPCR summaries of Cas9 edited HSPCs targeting *CD34* with PolQi2, AZD7648, and an ssODN donor template 3-, 7- and 14-days post editing. Data are shown as mean ± s.d. (*n*=3 technical replicates). Related to Fig. 3c. (**d**) End trimming summary of *CD34* edited HSPCs with PolQi2, AZD7648, and ssODN donor 3-, 7- and 14-days post editing. Data are shown as mean ± s.d. (*n*=3 technical replicates. Related to Fig. 3c. (**e**) NGS quantified repair profile of *CD34* edited iPSCs and HSPCs 3-, 7-, and 14-day post editing. Related to Fig. 3c. (**f**) Comparison of CLEAR-time dPCR and NGS editing summaries at the *CD34* locus in HSPCs and iPSCs 1-day post editing. Related to Fig. 3c. (**g**) CLEAR-time dPCR genome copy summary breakdown of Cas9 edited T cells (left) HSPCs (middle) and iPSCs (right) targeting the *CCR5* locus with and without AAV transduction and AZD7648 treatment 4-days post-editing. Statistical analysis performed on CLEAR-time dPCR data only. Two-way ANOVA with Tukey post-hoc test. *p<0.05, **p<0.01, ***p<0.001, ****p<0.0001. Targeted integration analysed using unpaired t-test. ****p<0.0001. Related to Fig. 3e.

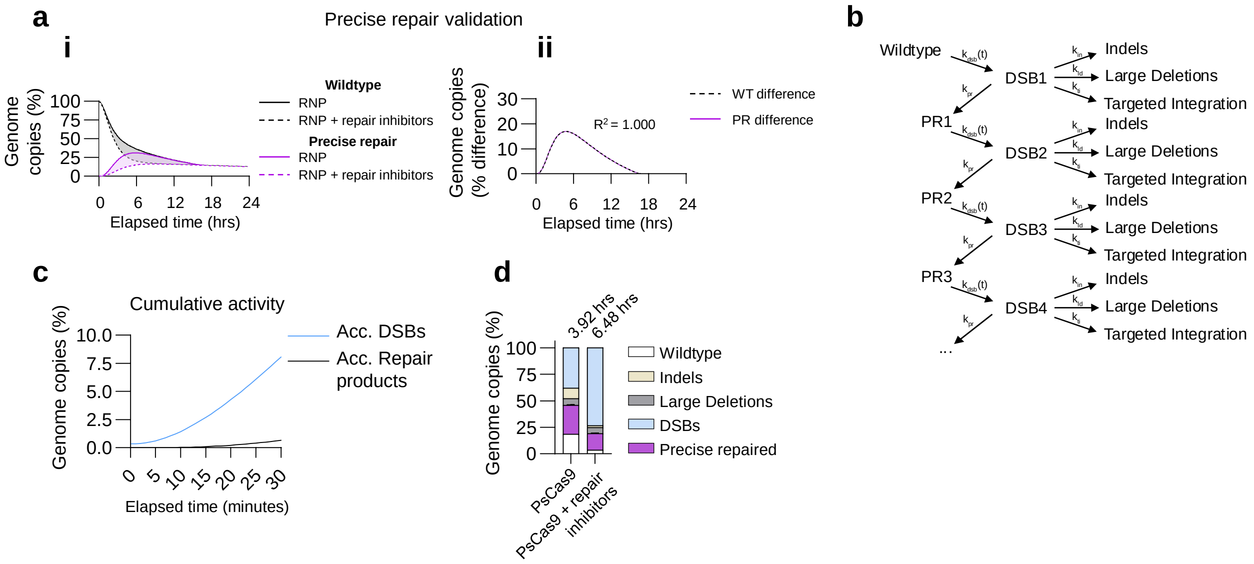

Supplementary Figure 4 | High-sensitivity kinetics of nuclease induced DSBs and repair

Precise repair validation (**i**) Comparison of the differences in simulated precise repair and fitted wildtype populations between RNP and RNP + repair inhibitor treatment groups, represented by the shaded regions between the curves. (**ii**) Overlap of the shaded WT and PR regions shown in (i), highlighting the accuracy of the k_{pr} estimation. (**b**) Model of recursive application of rate coefficients to the declining pool of uncleaved sequences. (**c**) Comparison between modelled accumulation of DSBs and repair products (sum of precise repair, indels, large deletions) in the first 30 minutes post-editing illustrating the speed in which repair occurs after initial DNA cleavage. Lines shown as the mean of 1000 bootstraps from *n*=3 technical replicates. (**d**) Bar chart illustrating genome copy frequency derived from modelling including the precise-repair frequency at the time at which DSB frequency peaked (time depicted above bars) in PsCas9 and PsCas9 + repair inhibitor treatment conditions.

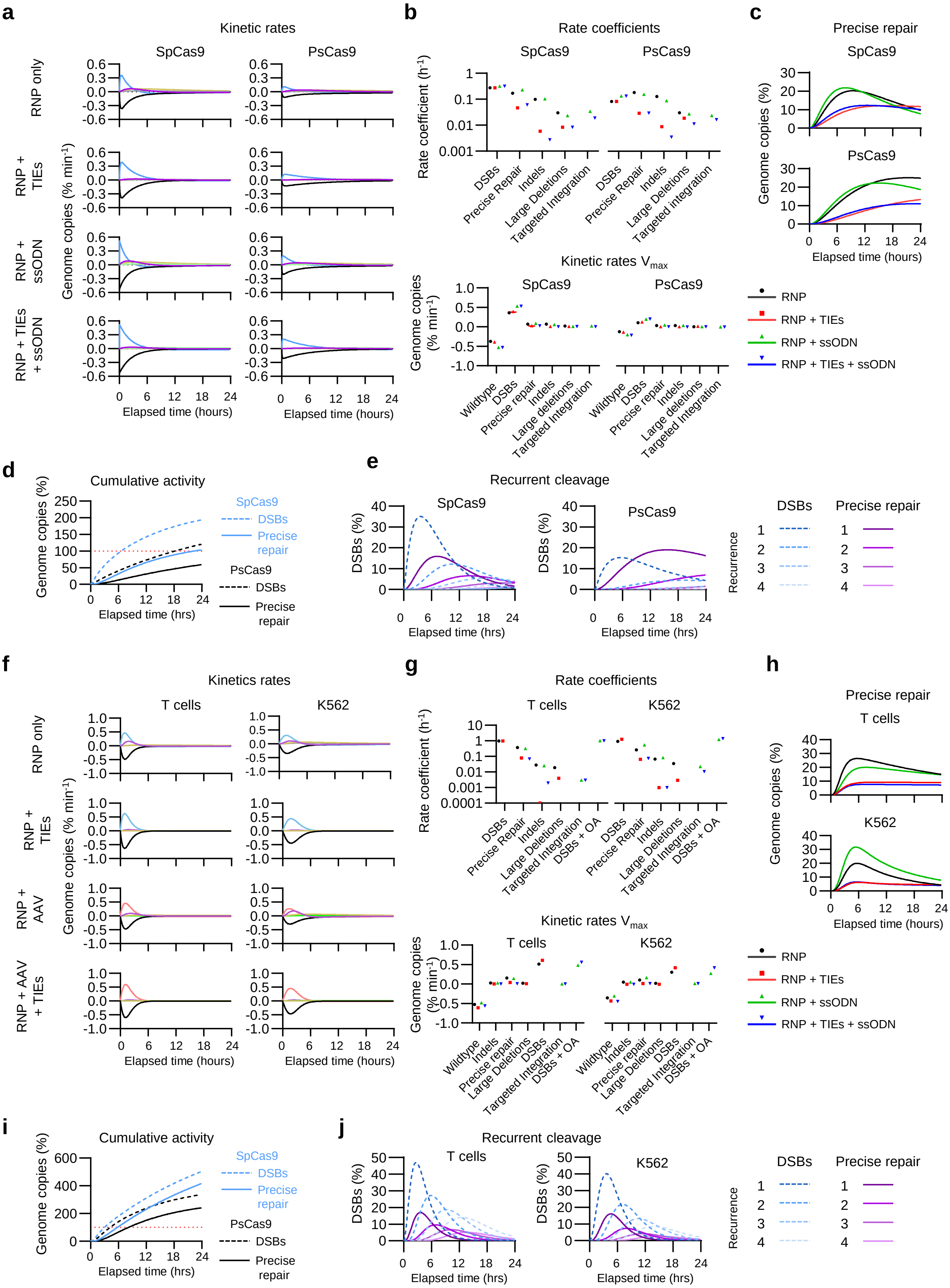
a
Kinetic rates
SpCas9
PsCas9
RNP only
RNP + TIEs
RNP + ssODN
RNP + TIEs + ssODN
Genome copies (% min-1)
Elapsed time (hours)
b
Rate coefficients
SpCas9
PsCas9
Rate coefficient (h-1)
DSBs
Precise Repair
Indels
Large Deletions
Targeted Integration
Kinetic rates Vmax
Genome copies (% min-1)
Wildtype
DSBs
Precise repair
Indels
Large deletions
Targeted Integration
c
Precise repair
SpCas9
PsCas9
Genome copies (%)
Elapsed time (hours)
RNP
RNP + TIEs
RNP + ssODN
RNP + TIEs + ssODN
d
Cumulative activity
Genome copies (%)
SpCas9
DSBs
Precise repair
PsCas9
DSBs
Precise repair
Elapsed time (hrs)
e
Recurrent cleavage
SpCas9
PsCas9
DSBs (%)
Elapsed time (hrs)
Recurrence
DSBs
Precise repair
f
Kinetics rates
T cells
K562
RNP only
RNP + TIEs
RNP + AAV
RNP + AAV + TIEs
Genome copies (% min-1)
Elapsed time (hours)
g
Rate coefficients
T cells
K562
Rate coefficient (h-1)
DSBs
Precise Repair
Indels
Large Deletions
Targeted Integration
DSBs + OA
Kinetic rates Vmax
Genome copies (% min-1)
Wildtype
Indels
Precise repair
Large Deletions
DSBs
Targeted Integration
DSBs + OA
h
Precise repair
T cells
K562
Genome copies (%)
Elapsed time (hours)
RNP
RNP + TIEs
RNP + ssODN
RNP + TIEs + ssODN
i
Cumulative activity
Genome copies (%)
SpCas9
DSBs
Precise repair
PsCas9
DSBs
Precise repair
Elapsed time (hrs)
j
Recurrent cleavage
T cells
K562
DSBs (%)
Elapsed time (hrs)
Recurrence
DSBs
Precise repair

Supplementary Figure 5 | Designer nuclease cleavage and repair kinetics in human primary cells and cell lines

(**a**) Kinetic activity rates per minute derived from the ODE fitted curves across all treatments in SpCas9 (left) and PsCas9 (right). (**b**) Comparison of rate coefficients, in events per hour, estimated by the ODE kinetics across all treatment groups for SpCas9 and PsCas9 edited cells. Related to Fig. 5b. (**c**) Comparison of precisely repaired DSBs across treatment groups for SpCas9 (top) and PsCas9 (bottom). (**d**) Accumulation of DSBs and precisely repaired sequences in RNP-only edited cells in a 24-hour time-period. (**e**) Model of recurring cleavage activity of precisely repaired DSBs in RNP only edited cells. Blue and purple lines indicate frequency of DSBs and precise repairs, respectively. Data shown as mean of 1000 bootstrapped estimations of k_{dsb} and k_{pr} coefficients used to infer genome copy frequencies from n=3 dPCR replicates. (**f**) Kinetic activity rates per minute derived from the ODE fitted curves across all treatments in T cells (left) and K562 cells (right). (**g**) Comparison of rate coefficients, in events per hour, estimated by the ODE kinetics across all treatment groups in both cell types tested. (**h**) Comparison of precisely repaired DSBs across treatment groups for T cells (top) and K562 cells (bottom). Related to Fig. 5g. (**i**) Accumulation of DSBs and precisely repaired DSBs in RNP-only edited cells in a 24-hour time-period. (**j**) Model of recurring DSB cleavage activity of precisely repaired DSBs in RNP only edited cells. Blue and purple lines indicate frequency of DSBs and precise repairs, respectively. Data shown as mean of 1000 bootstrapped estimations of k_{dsb} and k_{pr} coefficients used to infer genome copy frequencies from n=3 dPCR replicates. All data derived from modelling is shown as mean ± upper/lower limits of 95% CI (*n*=3 replicates, 1000 bootstraps per condition).

CLEAR-time dPCR equations

Copy number normalisations

To quantify the absolute frequency of these aberrations within the total population of alleles, copies derived from the FAM and HEX signals of the mutation assays were normalised (Norm 1) to the average number of reference assay copies amplified from two chromosomes unrelated to the edited one (Fig. 1b; Eq. 1). This initial normalisation to the reference was performed on unedited and edited cells.

$$\text{Norm 1} = \frac{\text{Assay copies per µl}}{\text{Reference copies per µl}}$$

Equation (1)

A second normalisation (Norm 2) was then performed on the relative value coming from the control unedited sample (Fig. 1b; Eq. 2).

$$\text{Norm 2} = \frac{\text{Edited Norm 1 assay}}{\text{Unedited Norm 1 assay}}$$

Equation (2)

Linkage equations

The percentage of linked sequences was calculated at the on-target site with the Flanking assay and also with the non-targeted chromosome via the *Reference* assay. First, the probability of two unlinked sequences co-segregating into the same droplet by chance (CD) and the sum of linked and unlinked 5’ and 3’ sequences (λ5’ and λ3’, respectively) were calculated.

We calculated the chance of co-segregating sequences by multiplying the number of FAM and HEX single droplets together and dividing by the total number of negative droplets (Eq. 3).

$$C_D = \frac{S_F \times S_H}{N_D}$$

Equation (3)

The sum of the linked and unlinked 5' sequences was calculated by subtracting the natural log of the FAM negative droplets from the natural log of the accepted droplets (Eq. 4).

$$\lambda 5' = ln(A_D) - ln(N_F)$$

Equation (4)

This was repeated for the 3' linked and unlinked sequences (Eq. 5).

$$\lambda 3' = ln(A_D) - ln(N_H)$$

Equation (5)

Linkage percentage was calculated by subtracting the natural log of the sum of the FAM single droplets, HEX single droplets, negative droplets, and chance co-segregated sequences from the natural log of the accepted droplets, multiplied by two, dividing this by the sum of the 5' and 3' linked and unlinked sequences, and finally multiplying by 100 (Eq. 6).

$$\% \text{ Linkage} = \frac{2\,(ln(A_D) - ln(S_F + S_H + N_D + C_D))}{\lambda 5' + \lambda 3'} \times 100$$

Equation (6)

Where;

C_D = Chance co-segregation

S_F = FAM single

S_H = HEX single

N_D = Negative droplets

A_D = Accepted droplets

N_F = FAM negative droplets

N_H = HEX negative droplets

λ5’ = sum of 5’ linked and unlinked sequences

λ3’ = sum of 3’ linked and unlinked sequences

Linkage normalisations

The linkage of the on-target flanking assay was normalised (Norm 3) to the linkage of the references for both the unedited and edited cells (Fig. 1b; Eq. 7).

$$\text{Norm 3} = \frac{\%\ \text{Flanking linkage}}{\%\ \text{Reference linkage}}$$

Equation (7)

The reference normalised linkage of the edited cells was then normalised (Norm 4) to the reference normalised linkage of the unedited cells (Fig. 1b; Eq. 8).

$$\text{Norm 4} = \frac{\text{Edited Norm 3 linkage}}{\text{Unedited Norm 3 linkage}}$$

Equation (8)

Donor template normalisation

When using a donor template where the length of the homology arms is excessively greater than the reference assay, a size-matched reference should be deployed to account for quantification bias. The number of integration copies should be normalised (Norm 5) to the number of size-matched reference copies (Fig. 1b; Eq. 9).

$$\text{Norm 5} = \frac{\text{Targeted integration copies per μl}}{\text{Size-matched reference copies per μl}}$$

Equation (9)

As no integration occurs in unedited cells, there is no requirement to normalise to this control.

Allele frequencies

To calculate the percentage of wildtype alleles, the Norm 2 Edge assay FAM (cleavage) value was multiplied by 100 (Fig. 1b; Eq. 10).

$$\%\ \mathrm{Wildtype} = \mathrm{Norm\ 2\ Edge\ FAM} \times 100$$

Equation (10)

The Indel percentage was calculated by subtracting the Norm 2 Edge assay HEX (distal) value from the Norm 2 Edge assay FAM value and multiplying by 100 (Fig. 1b; Eq. 11).

$$\%\ \mathrm{Indel} = (\mathrm{Norm\ 2\ Edge\ FAM} - \mathrm{Norm\ 2\ Edge\ HEX}) \times 100$$

Equation (11)

We then calculated the total non-indel aberrations by subtracting 1 from the Norm 2 Edge assay HEX value and multiplying by 100 (Fig. 1b; Eq. 12).

$$\%\ \mathrm{Total\ non\text{-}indel\ aberrations} = (\mathrm{Norm\ 2\ Edge\ HEX} - 1) \times 100$$

Equation (12)

We calculated the percentage of linkage loss by subtracting 1 from the Norm 4 linkage value and multiplying by 100 (Fig. 1b; Eq. 13).

$$\%\ \mathrm{Linkage\ loss} = (\mathrm{Norm\ 4} - 1) \times 100$$

Equation (13)

No loss of linkage would be observed in the case of extended bidirectional end resection; therefore, the difference between the non-indel aberrations detected by the edge assay and the linkage loss quantified from the flanking assay was calculated as a large deletion (Fig. 1b; Eq. 14).

$$\%\ \text{Large deletions} = \%\ \text{Non-indel aberrations} - \%\ \text{Linkage loss}$$

Equation (14)

The percentage of linkage loss was then defined as “other aberrations,” i.e., double-strand breaks, translocations, inversions, chromothripsis, etc. (Fig. 1b; Eq. 15).

$$\%\ \text{Other aberrations} = \%\ \text{Linkage loss}$$

Equation (15)

We calculate the integration frequency for autosomes and X-linked genes in XX cells by multiplying the Norm 5 targeted integration value by 100 (Eq. 16).

$$\%\ \text{Targeted integration (Autosomes)} = \text{Norm}\ 5\ \times 100$$

Equation (16)

Whereas X- or Y-linked genes in XY cells, integration frequency is calculated by first multiplying the Norm 5 targeted integration value by 2 to account for half the alleles that can be integrated, then again by 100 (Fig. 1b; Eq. 17).

$$\%\ \text{Targeted integration (sex-linked XY)} = \text{Norm}\ 5\ \times 2\ \times 100$$

Equation (17)

The flanking assay is unable to differentiate endogenous sequences and donor templates with long homology arms and, thus, in this circumstance, it is incapable of calculating large

deletions. We calculate the resulting aberrations by subtracting the targeted integration from the total non-indel aberrations (Fig. 1b; Eq. 18).

$$\text{\% Large deletions|Other aberrations} = \text{\% Total non-indel aberrations} - \text{\% Targeted integration}$$

Equation (18)

Modelling ODE equations

$$WT\ rate = dWT/dt = (k_{pr} \times DSB) - (k_{dsb} \times D(t) \times WT)$$

$$DSB\ rate = dDSB/dt = (k_{dsb} \times D(t) \times WT) - (k_{pr} + k_{in} + k_{ld} + k_{ti}) \times DSB$$

$$Precise\ repair\ rate = dPR/dt = (k_{pr} \times DSB) - (k_{dsb} \times D(t) \times PR)$$

$$Indel\ rate = dIndel/dt = (k_{in} \times DSB)$$

$$Large\ deletions\ rate = dLD/dt = (k_{ld} \times DSB)$$

$$Targeted\ integration\ rate = dTI/dt = (k_{ti} \times DSB)$$

$$D(t) = 1 - 2^{-(t/delay)}$$

Equation (19)

Where;

d[allele]/dt = difference in allele frequency/difference in time (rate)

k_{dsb}, k_{pr}, k_{in}, k_{ld}, k_{ti}, = rate coefficients of DSBs, precise repair, indels, large deletions, and targeted integration, per hour respectively.

D(t) = Cas9 nuclear trafficking delay based on the time in hours (t).

Rate coefficient derived information

We implemented the rate coefficients derived from the ODE modelling in established decay rate kinetics equations to derive information regarding the time for half of the wildtype alleles to deplete and DSBs to resolve.

$$\text{DSB generation half-life (hrs)} = \frac{ln2}{\mathrm{k_{dsb}}}$$

Equation (20)

$$\text{DSB resolution half-life (hrs)} = \frac{ln2}{\mathrm{k_{pr}} + \mathrm{k_{in}} + \mathrm{k_{ld}} + \mathrm{k_{ti}}}$$

Equation (21)

Using ratios of the rate coefficients, we also calculated the likelihood of a specific repair product forming (or remaining unresolved) within an hour of the DSB being generated, and the number of DSBs to have occurred before a specific repair product is generated.

$$\text{Repair likelihood (\%)} = \frac{\text{Repair product (k) or unresolved DSBs}}{\mathrm{k_{pr}} + \mathrm{k_{in}} + \mathrm{k_{ld}} + \mathrm{k_{ti}}}$$

Regarding unresolved DSBs, if $\mathrm{k_{dsb}} - (\mathrm{k_{pr}} + \mathrm{k_{in}} + \mathrm{k_{ld}} + \mathrm{k_{ti}}) < 0$, then replace with 0. (i.e., no unresolved DSBs).

Equation (22)

$$\text{DSBs per repair} = \frac{Sum\ of\ repair\ products\ (k)}{Repair\ product\ (k)}$$

Equation (23)
