# Supplementary material for: Unveiling the cut-and-repair cycle of designer nucleases in human stem and T cells via CLEAR-time dPCR
Source: Nat Commun. 2025 Nov 3;16:9571. doi: 10.1038/s41467-025-65182-4 (PMC12583642; doi:10.1038/s41467-025-65182-4)
Supplement: Supplementary file 2 — Description of Additional Supplementary Files [file 41467_2025_65182_MOESM2_ESM.pdf]

Description of Additional Supplementary Files

File Name: Supplementary Data 1
Description: Table 1 | Primer and Probes

File Name: Supplementary Data 2
Description: Table 2 | sgRNA sequences

File Name: Supplementary Data 3
Description: Table 3 | Donor template sequence

File Name: Supplementary Data 4
Description: Table 4 | PCR conditions

File Name: Supplementary Data 5
Description: Table 5 | Materials
