# Supplementary material for: Unveiling the cut-and-repair cycle of designer nucleases in human stem and T cells via CLEAR-time dPCR
Source: Nat Commun. 2025 Nov 3;16:9571. doi: 10.1038/s41467-025-65182-4 (PMC12583642; doi:10.1038/s41467-025-65182-4)
Supplement: Supplementary file 8 — Reporting Summary [file 41467_2025_65182_MOESM8_ESM.pdf]

nature portfolio

Corresponding author(s): Giandomenico Turchiano

Last updated by author(s): May 23, 2025

Reporting Summary

Nature Portfolio wishes to improve the reproducibility of the work that we publish. This form provides structure for consistency and transparency in reporting. For further information on Nature Portfolio policies, see our Editorial Policies and the Editorial Policy Checklist.

Statistics

For all statistical analyses, confirm that the following items are present in the figure legend, table legend, main text, or Methods section.

n/a	Confirmed	
☐	☒	The exact sample size (n) for each experimental group/condition, given as a discrete number and unit of measurement
☐	☒	A statement on whether measurements were taken from distinct samples or whether the same sample was measured repeatedly
☐	☒	The statistical test(s) used AND whether they are one- or two-sided<br>*Only common tests should be described solely by name; describe more complex techniques in the Methods section.*
☒	☐	A description of all covariates tested
☐	☒	A description of any assumptions or corrections, such as tests of normality and adjustment for multiple comparisons
☐	☒	A full description of the statistical parameters including central tendency (e.g. means) or other basic estimates (e.g. regression coefficient) AND variation (e.g. standard deviation) or associated estimates of uncertainty (e.g. confidence intervals)
☐	☒	For null hypothesis testing, the test statistic (e.g. F, t, r) with confidence intervals, effect sizes, degrees of freedom and P value noted<br>*Give P values as exact values whenever suitable.*
☒	☐	For Bayesian analysis, information on the choice of priors and Markov chain Monte Carlo settings
☒	☐	For hierarchical and complex designs, identification of the appropriate level for tests and full reporting of outcomes
☒	☐	Estimates of effect sizes (e.g. Cohen's d, Pearson's r), indicating how they were calculated

Our web collection on statistics for biologists contains articles on many of the points above.

Software and code

Policy information about availability of computer code

Data collection	1) QuantaSoft (Bio-Rad) was used to analyze ddPCR<br>2) BD FACSDIVA (Becton Dickinson) software was included in the BD FACSaria II and BD LSR II analyzer and was used to collect flow cytometry data<br>3) IGV genome browser visualisation
Data analysis	1) PRISM software. for statistical analysis and plotting<br>2) kinetics modelling avaialble at: github.com/A969 Chalk/MEGA and github.com/A-Chalk/DSB-Kinetics-Calculator respectively.<br>3) CAST-seq testing available at: https://github.com/AG-Boerries/CAST-Seq<br>4) CRISPRESSO2/RIMA pipeline for NGS deep sequencing data.<br>5) ICE (synthego)<br>6) Excel microsoft<br>7) ImageJ<br>8) SnapGene

For manuscripts utilizing custom algorithms or software that are central to the research but not yet described in published literature, software must be made available to editors and reviewers. We strongly encourage code deposition in a community repository (e.g. GitHub). See the Nature Portfolio guidelines for submitting code & software for further information.

Data

Policy information about availability of data

All manuscripts must include a data availability statement. This statement should provide the following information, where applicable:

- Accession codes, unique identifiers, or web links for publicly available datasets
- A description of any restrictions on data availability
- For clinical datasets or third party data, please ensure that the statement adheres to our policy

Raw data presented in the source data tabular file.

Research involving human participants, their data, or biological material

Policy information about studies with human participants or human data. See also policy information about sex, gender (identity/presentation), and sexual orientation and race, ethnicity and racism.

Reporting on sex and gender	*Use the terms sex (biological attribute) and gender (shaped by social and cultural circumstances) carefully in order to avoid confusing both terms. Indicate if findings apply to only one sex or gender; describe whether sex and gender were considered in study design; whether sex and/or gender was determined based on self-reporting or assigned and methods used. Provide in the source data disaggregated sex and gender data, where this information has been collected, and if consent has been obtained for sharing of individual-level data; provide overall numbers in this Reporting Summary. Please state if this information has not been collected. Report sex- and gender-based analyses where performed, justify reasons for lack of sex- and gender-based analysis.*
Reporting on race, ethnicity, or other socially relevant groupings	*Please specify the socially constructed or socially relevant categorization variable(s) used in your manuscript and explain why they were used. Please note that such variables should not be used as proxies for other socially constructed/relevant variables (for example, race or ethnicity should not be used as a proxy for socioeconomic status). Provide clear definitions of the relevant terms used, how they were provided (by the participants/respondents, the researchers, or third parties), and the method(s) used to classify people into the different categories (e.g. self-report, census or administrative data, social media data, etc.) Please provide details about how you controlled for confounding variables in your analyses.*
Population characteristics	*Describe the covariate-relevant population characteristics of the human research participants (e.g. age, genotypic information, past and current diagnosis and treatment categories). If you filled out the behavioural & social sciences study design questions and have nothing to add here, write "See above."*
Recruitment	*Describe how participants were recruited. Outline any potential self-selection bias or other biases that may be present and how these are likely to impact results.*
Ethics oversight	*Identify the organization(s) that approved the study protocol.*

Note that full information on the approval of the study protocol must also be provided in the manuscript.

Field-specific reporting

Please select the one below that is the best fit for your research. If you are not sure, read the appropriate sections before making your selection.

☒ Life sciences ☐ Behavioural & social sciences ☐ Ecological, evolutionary & environmental sciences

For a reference copy of the document with all sections, see nature.com/documents/nr-reporting-summary-flat.pdf

Life sciences study design

All studies must disclose on these points even when the disclosure is negative.

Sample size	Triplicate experiments were performed to minimize technical variabilities. Multiple independent samples and/or targets were tested to confirm technical robustness of the analysis providing supporting orthogonal methods data to confirm the given observation. The number of animals used per transplant were determined by the number of cells successfully modified from a single human source divided by the intended dose. The minimum number of mice per condition was determined through a pilot study which provided us with information about the standard deviation and the magnitude of the effects. In addition, the sample size used in this experiment is consistent with previous reports (Pavel-Dinu et al., 2019, Nature Comm; Gomez-Ospina et al., 2019, Nature Comm).
Data exclusions	No datasets were excluded from this work.
Replication	Triplicate experiments were performed to minimize to technical variabilities.
Randomization	Not relevant for this study. Most of the dPCR analyses requires a previous knowledge of the control/unedited samples for data normalisation.
Blinding	No blinding procedure required as the observations were not dependent to an outcome or foreseeable.

Reporting for specific materials, systems and methods

We require information from authors about some types of materials, experimental systems and methods used in many studies. Here, indicate whether each material, system or method listed is relevant to your study. If you are not sure if a list item applies to your research, read the appropriate section before selecting a response.

Materials & experimental systems

n/a	Involved in the study
☐	☒ Antibodies
☐	☒ Eukaryotic cell lines
☒	☐ Palaeontology and archaeology
☐	☒ Animals and other organisms
☒	☐ Clinical data
☒	☐ Dual use research of concern
☒	☐ Plants

Methods

n/a	Involved in the study
☒	☐ ChIP-seq
☐	☒ Flow cytometry
☒	☐ MRI-based neuroimaging

Antibodies

Antibodies used	Mouse anti-human CD45APC HI30 BD Bioscience 555485<br>Antimouse CD45 BV605 clone 30-F11 BD-biolegend<br>CD34 MicroBead Kit (Miltenyi Bioscience)
Validation	All the antibodies used in this study were routinely tested in flow cytometry. Antibodies were validated by using positive (antigen positive cells) and negative (isotype and/or antigen negative cells) controls with the recommended antibody concentrations from the manufacturers.

Eukaryotic cell lines

Policy information about cell lines and Sex and Gender in Research

Cell line source(s)	Informed written consent was obtained from healthy donors for the use of human CD34+ haematopoietic stem and progenitor cells (HSPCs), in accordance with the Declaration of Helsinki. Ethical approval was granted by the Great Ormond Street Hospital for Children NHS Foundation Trust and the Institute of Child Health Research Ethics Committee (08/H0713/87). Lonza (HSPC), Great Ormond Street Hospital apharesis (HSPC, T-cells). K562 cells obtained from ATCC CRL-243. AAVpro® 293T Cell Line obtained from Takara cat# 632273.
Authentication	Morphological and flow cytometry testing.
Mycoplasma contamination	The cell line tested negative for mycoplasma contamination
Commonly misidentified lines<br>(See ICLAC register)	Nocommonly misidentified cell lines were used in this study

Animals and other research organisms

Policy information about studies involving animals; ARRIVE guidelines recommended for reporting animal research, and Sex and Gender in Research

Laboratory animals	Mus musculus NSG (NOD.CB17-PRKDCSCID/j); 8 weeks old engraftment time. Culled 16 weeks after the xenotransplantation.
Wild animals	No wild animals utilised
Reporting on sex	Only female mice were utilised to minimise the sex engraftment variation.
Field-collected samples	No field collected animals involved in this study.
Ethics oversight	University College London Animal Welfare and Ethical Review Body. Project license 70/8241

Note that full information on the approval of the study protocol must also be provided in the manuscript.

Plants

Seed stocks | *Report on the source of all seed stocks or other plant material used. If applicable, state the seed stock centre and catalogue number. If plant specimens were collected from the field, describe the collection location, date and sampling procedures.*

Novel plant genotypes | *Describe the methods by which all novel plant genotypes were produced. This includes those generated by transgenic approaches, gene editing, chemical/radiation-based mutagenesis and hybridization. For transgenic lines, describe the transformation method, the number of independent lines analyzed and the generation upon which experiments were performed. For gene-edited lines, describe the editor used, the endogenous sequence targeted for editing, the targeting guide RNA sequence (if applicable) and how the editor was applied.*

Authentication | *Describe any authentication procedures for each seed stock used or novel genotype generated. Describe any experiments used to assess the effect of a mutation and, where applicable, how potential secondary effects (e.g. second site T-DNA insertions, mosiacism, off-target gene editing) were examined.*

Flow Cytometry

Plots

Confirm that:

☒ The axis labels state the marker and fluorochrome used (e.g. CD4-FITC).

☒ The axis scales are clearly visible. Include numbers along axes only for bottom left plot of group (a 'group' is an analysis of identical markers).

☒ All plots are contour plots with outliers or pseudocolor plots.

☒ A numerical value for number of cells or percentage (with statistics) is provided.

Methodology

Sample preparation	Cells stained in PBS+ 1%BSA, incubated 30 minutes and washed before resuspension in PBS+1% BSA
Instrument	BD LSRII.
Software	BD FACS DIVA.<br>FlowJo
Cell population abundance	>10000 cells were analysed for each sample with sub-populations at >10% of the overall cell number
Gating strategy	viable populations gated from physical parameters FSC-H Vs FSC-W; then FSC-A Vs SSC. DAPI staining was defining the apoptotic cell population to exclude. viable cells were than gated for the marker studied utilising untreated samples to set up negative population thresholds.

☒ Tick this box to confirm that a figure exemplifying the gating strategy is provided in the Supplementary Information.
